# Molecular basis of the explosive defence response in the bombardier beetle *Brachinus crepitans*

**DOI:** 10.1098/rsos.241823

**Published:** 2025-05-21

**Authors:** Heiko Vogel, Nicolai Rügen, Natalie Wielsch, Richard M. Twyman, Miray Tonk-Rügen, Andreas Vilcinskas

**Affiliations:** ^1^ Department of Insect Symbiosis, Max Planck Institute for Chemical Ecology, Jena, Germany; ^2^ Branch for Bioresources, Fraunhofer Institute for Molecular Biology and Applied Ecology IME, Giessen, Hessen 35392, Germany; ^3^ Research Group Mass Spectrometry/Proteomics, Max-Planck-Institute for Chemical Ecology, Jena, Thüringen 07745, Germany; ^4^ TRM Ltd, Scarborough, UK; ^5^ Institute for Insect Biotechnology, Justus Liebig Universitat Giessen, Giessen, Hessen 35392, Germany

**Keywords:** chemical defence, omics, hydroquinone, hydrogen peroxide, bombardier beetles, *Brachinus crepitans*

## Abstract

Bombardier beetles have evolved a sophisticated and unique chemical defence mechanism involving controlled explosions within their paired defensive glands, producing a hot, benzoquinone-rich defensive spray. The molecular basis of this response is not well characterized. We therefore combined the transcriptomic and proteomic analysis of different gland compartments in the bombardier beetle *Brachinus crepitans* (Linnaeus, 1758) (Coleoptera, Carabidae) to identify abundant transcripts and gland-specific proteins with key defensive functions, such as catalases, peroxidases and enzymes involved in hydroquinone synthesis. By combining precise dissections with protein sequence analysis, we built a comprehensive atlas of the relevant proteins and their spatio-temporal organization. We found that glucose is important as a stable precursor of hydrogen peroxide and hydroquinone. These chemicals, together with gland-specific peroxidases and catalases, then initiate the explosive defence reaction. We also present evidence that the evolution of explosive secretions involved the functional adaptation of peroxidase genes involving atypical substitutions in otherwise highly conserved protein domains.

## 1. Introduction

**Figure 1.**
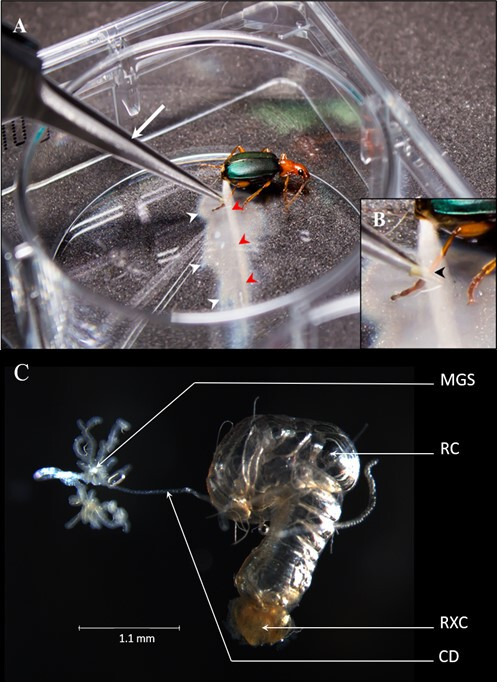
Explosive defence reaction of the bombardier beetle *Brachinus crepitans* and its dissected defensive glands. (A) The defence secretion is ejected as a jet of fine mist without visible dissipation (red arrowheads) in a rapid series of consecutive pulses. Part of the secretion was deflected during an earlier pulse onto the bottom of the plate, and is visible as a puddle (white arrowheads). (B) Higher magnification highlights the precision with which the beetle orientates the jet towards the contact point with forceps (black arrowhead). (C) Freshly dissected and untreated defensive glands of a female *B. crepitans* specimen. MGS = multi-lobed glandular system, CD = collection duct, RC = reservoir chamber, RXC = reaction chamber.

Many beetle species have independently evolved the ability to deploy chemical defence mechanisms against predators, competitors or even pathogens [1–5]. The most fascinating example is found among bombardier beetles (Carabidae; Brachininae), which forcibly expel a hot spray of noxious *p*-benzoquinones from their paired abdominal defensive glands when attacked by vertebrate or invertebrate predators [6–8] or when stimulated experimentally (figure 1A,B).

The anatomy, mechanics and biochemistry of the defensive glands are well characterized in the genus *Brachinus* [9–14]. There are three main elements: a multi-lobed glandular system (MGS) with a collection duct (CD), reservoir chamber (RC) and reaction chamber (RXC) adjacent to an exit channel that vents at the tip of the abdomen (figure 1C) [9]. Glandular secretions enter the CD and accumulate in the RC, which is surrounded by muscles [9,10]. When the beetle is provoked, these muscles contract to force the stored chemicals—an aqueous solution of approximately 25% hydrogen peroxide and approximately 10% *p*-hydroquinones along with approximately 10% *n*-alkanes as a non-reactive second liquid phase acting as a solvent [10]—through a one-way valve into the rigid-walled, sclerotized RXC lined with cells that are thought to secrete catalases and peroxidases, although this has not been confirmed. Mixing initiates an explosive exothermic reaction that produces *p*-benzoquinones, free oxygen and water, also generating 48.5 kcal of thermal energy per mol *p*-benzoquinones. The high overpressure in the RXC results in an explosive secretory discharge that cannot pass back through the valve into the RC [10]. It is therefore ejected as a noxious pulsed jet (figure 1A) in the direction of the perceived threat (figure 1B) at temperatures of up to 100°C, at speeds of up to 10 m s^−1^ and over distances of up to 30 cm [10–14]. The luminal surfaces of the chambers are lined with cuticular material to protect the underlying cells from the chemicals and heat [9,10], and are probably involved in the pulsed mechanism of the defensive reaction.

The subfamily Brachininae of the adephagan ground beetles (Carabidae) encompasses more than 500 species and is commonly known as bombardier beetles. They represent the only animal species known thus far that can sustain an explosion of hot chemicals within their body, a particularly remarkable feature given that two of the key ingredients—*p*-benzoquinones and hydrogen peroxide—are potent cytotoxins [15]. However, the molecular basis of the defence reaction has not been characterized in detail, and the gene products required in the different compartments are unknown. In particular, it is unclear how the hydroquinones are synthesized, how they are safely stored along with the hydrogen peroxide, how the *n*-alkanes in the RC are synthesized, which peroxidases and catalases trigger the explosive reaction, and how the system recharges after depletion. We therefore carried out a detailed transcriptomic and proteomic analysis of the defensive glands in the beetle *Brachinus crepitans* to map the gene expression, protein accumulation and candidate enzymes in each chamber and thus provide a comprehensive spatio-temporal description of how the defence reaction is controlled. Once the secretions are exhausted, the recharging of the defence system is presumably accompanied by the induced expression of genes that are required to reload the defensive glands. We therefore monitored the recharging time to determine the most appropriate sampling time points for transcriptomic analysis.

## 2. Material and methods

### 2.1. Beetle collection, maintenance and species identification

Adult bombardier beetles (*B. crepitans*) were collected from Nordrhein-Westfalen, Germany, with permission from the Nature Conservation Agency in Koblenz (ref. 425-104.1713). The beetles were maintained in terrariums filled with a mixture of autoclaved soil from the collection areas, plus limestone and hay as a covered oviposition substrate. The terrariums were humidified with tap water twice weekly. The beetles were kept together in populations of approximately 200 along with other beetle species from the same collection areas (*Anchomenus dorsalis* (Pontoppidan, 1763) (Coleoptera, Carabidae), *Poecilus cupreus* (Linnaeus, *1758*) (Coleoptera*,* Carabidae) and *Amara* spp.). In total, more than approximately 600 specimens of *B. crepitans* were collected. Before analysis, the beetles were adapted to laboratory conditions (20–22°C) for 1 year, and fed with mealworms (*Tenebrio molitor*) and fresh tap water every 2−3 days. All beetles used for the experiments were visually assessed for fitness by controlling their natural mobility when placed on a Petri dish during daylight, and by checking for the presence of macro-parasites under a stereomicroscope.

Bombardier beetles were identified using taxonomic descriptions [16] and by DNA sequencing [17]. Genomic DNA was isolated from the legs of two beetles using the Quick-DNA Tissue/Insect Microprep Kit according to the manufacturer’s instructions (Zymo Research, Germany). Polymerase chain reaction (PCR) was carried out using a C100 thermal cycler (BioRad, Germany) and Phusion Flash High-Fidelity PCR Master Mix (Thermo Fisher Scientific, Germany). Primers were designed against the *B. crepitans* reference gene *COI* (GenBank KU914043.1) using PrimerQuestTool (Integrated DNA Technologies, Germany) and were synthesized by Eurofins Genomics (Germany). The sequences of the forward and reverse primers were 5′-GCA GGA ATA GTA GGG ACT T-3′ and 5′-GCA GGA ATA GTA GGG ACT T-3′, respectively. Each reaction was heated to 98°C and held for 10 s before 32 cycles of 98°C for 1 s, 60°C for 5 s and 72°C for 5 s and a final extension step at 72°C for 1 min. Following agarose gel electrophoresis, the two anticipated bands were isolated and the DNA was recovered using a Zymoclean purification kit (Zymo Research) for sequencing using a Mix2Seq kit (Eurofins Genomics). The resulting sequences (GenBank EF00497196 and EF00497197) were used as BLASTN queries, confirming the highest percentage identity with *B. crepitans COI*.

### 2.2. Duration of gland recharging after treatment

Before dissecting the beetles for transcriptome sequencing, we determined the optimum time points for maximum transcript levels at the time of dissection by measuring the time taken for beetles to recharge their glands after defence spray exhaustion (electronic supplementary material, figure S1). Ten beetles were placed individually in six-well plates (Sigma-Aldrich, Germany). At time T_0_ and at hourly intervals thereafter, the defence response was provoked by repeatedly pinching the hind legs with forceps until the spray stopped. The number of spray events during treatment was counted to determine the duration required for the beetles to recharge their defensive glands.

### 2.3. Sample preparation and RNA isolation for transcriptome analysis

Beetles were separated into groups of 24 and were placed individually into the wells of six-well plates. One group was treated at time T0 and hourly thereafter by pinching the legs with forceps to encourage spraying and exhaustion of the defensive secretions, and was anaesthetized after 6 h once the reservoirs were fully depleted by pre-chilling at 4°C for 30 min before dissection. The other group was left untreated as a control and was chilled and dissected immediately (T_0_). The entire defensive glands were separated from the residual body tissues in pre-chilled (4°C) phosphate-buffered saline (PBS) followed by flash-freezing in liquid nitrogen and storage at −80°C. The dissected defensive glands comprised the MGS, CD, RC and RXC. Total RNA was isolated from the defensive glands and the body residues without glands of untreated and provoked beetles using the ZR Tissue & Insect RNA MicroPrep Kit (Zymo Research) according to the manufacturer’s instructions. The quantity and quality of the RNA were determined by measuring the optical density at 260 and 280 nm with an Eon microplate spectrophotometer (BioTek Instruments, USA) and using the RNA 6000 Nano Kit on an Agilent Bioanalyzer (Agilent Technologies, USA).

### 2.4. RNA-Seq, de novo assembly and candidate gene identification

Transcriptome sequencing was carried out by GATC Biotech using poly(A)^+^ enriched RNA fragmented to an average of 220 nucleotides. We used the Illumina HiSeq2500 Genome Analyzer platform and paired-end (2 × 150 bp) read technology, yielding 40−50 million reads for each of the four samples (treated and untreated defensive glands and body residues). Quality controls and three de novo transcriptome assemblies, using only the gland or body sample data, as well as data from all samples combined, were prepared using CLC Genomics Workbench v. 11.1 (http://www.clcbio.com) as previously described [18,19]. The de novo reference transcriptome assemblies of *B. crepitans* contained 30 565 contigs for the gland-only assembly, 45 201 contigs for the rest of body-only assembly and 50 332 contigs for the combined assembly (minimum contig size = 250 bp), with N50 contig sizes of 1894, 1737 and 1709 bp and maximum contig lengths of 16 294, 26 132 and 27 953 bp, respectively. Transcriptome annotation using BLAST, Gene Ontology and InterProScan was carried out as previously described [19]. BUSCO analysis (http://busco.ezlab.org) using a set of highly conserved single-copy orthologues as a reference was carried out using the BUSCO v. 3 pipeline [20]. We compared the proteins predicted from the *B. crepitans* transcriptomes to the predefined set of 1658 Insecta single-copy orthologues from the OrthoDB v. 9.1 database. This indicated completeness values of 86.3, 90.1 and 90.9% for the gland-only, rest of body-only and combined assemblies respectively, with 9.3, 6.2 and 6% missing BUSCO genes.

Transcripts from the gland-only, rest of body-only and combined assemblies that were annotated as catalases, peroxidases, glucose dehydrogenases (GDH) or glucosylceramidases were confirmed by six-frame translation using the Expasy translation tool (https://web.expasy.org/translate/) followed by BLAST searches of the UniProt database. The domain architecture of the sequences from the transcriptome dataset was assessed using the integrated InterProScan tool [21]. Signal peptide cleavage sites were predicted with SignalP v. 5.0 (https://services.healthtech.dtu.dk/services/SignalP-5.0/).

### 2.5. Mapping and differential gene expression analysis

Digital gene expression analysis was carried out using CLC Genomics Workbench v. 12.0.2 to generate binary alignment map (BAM) mapping files and by counting the sequences to estimate expression levels using previously described parameters for read mapping and normalization [18,19]. The log_2_ transcripts per million (TPM) values (normalized mapped read values) were subsequently used to calculate fold-change (FC) values between samples to compare treated versus untreated and glands versus body datasets. To test for the effect of different normalization methods, mapped reads were normalized and transformed with generalized linear models using the empirical analysis of digital gene expression (EDGE) tool in CLC Genomics Workbench v. 12.0.2, allowing for a non-constant mean–variance relationship in the read count data. Differential gene expression analysis between control and treated (spray-depleted) samples was conducted using the NOISeq R/Bioc package [22] in Omicsbox v. 2.1.

### 2.6. Data submission

Illumina short reads have been deposited in the EBI short read archive (SRA) with the following sample accession numbers: ERS12235361, ERS12235368–ERS12235370. The complete study can also be accessed directly using the following URL: http://www.ebi.ac.uk/ena/data/view/PRJEB53429. The short-read data and electronic supplementary material, files S1–S3 have been submitted to the Edmond database with the following URLs: https://doi.org/10.17617/3.YODKCZ (RNA-Seq data), https://doi.org/10.17617/3.WO4CVD (gland transcriptomic data, annotation and gene expression analysis; electronic supplementary material, file S1), https://doi.org/10.17617/3.VASY8P (combined transcriptomic data, annotation and gene expression analysis; electronic supplementary material, file S2) and https://doi.org/10.17617/3.EIZ5MG (proteomics data; electronic supplementary material, file S3).

### 2.7. Proteomics sample preparation and analysis by liquid chromatography–mass spectrometry

Defensive spray was collected by provoking the beetles with forceps and mixed with 200 µl PBS containing 1× Pierce protease inhibitor cocktail before storage at −80°C. Tissue samples (RC, RXC and MGS/CD) were collected on ice in 30 µl PBS containing 1× Pierce protease inhibitor cocktail, gently squeezed with a small pistil and centrifuged at 5000*g* for 5 min at 4°C to pellet any particulate matter. Supernatants were mixed with XT sample loading buffer and reducing agent (BioRad), incubated at 98°C for 5 min to denature the proteins and fractionated by SDS-PAGE on a Criterion XT Bis-Tris 4−12% polyacrylamide gradient gel (BioRad) with XT MES running buffer. The gel was stained with Coomassie Brilliant Blue (PageBlue protein staining solution, Thermo Fisher Scientific). Thirty gel segments for each sample, representing different protein size ranges, were excised and digested with trypsin as previously described [23]. The tryptic peptides were extracted and reconstructed in 50 μl aqueous 0.1% formic acid.

For liquid chromatography with tandem mass spectrometry (LC-MS/MS) analysis, 1 µl of the peptide mixture was fractionated on an M-class ultra-performance liquid chromatography (UPLC) system coupled online to a Synapt G2-si mass spectrometer equipped with a T-WAVE-IMS device (Waters, Germany). The samples were pre-concentrated and desalted on an M-Class Symmetry C18 trap column (100 Å, 180 µm × 20 mm, 5 µm particle size) at a flow rate of 15 µl min^−1^ (0.1% aqueous formic acid), and the peptides were eluted onto an ACQUITY UPLC HSS T3 analytical column (100 Å, 75 μm × 200, 1.8 μm particle size) at a flow rate of 350 nl min^−1^ in a mixture of buffers A (0.1% formic acid in water) and B (0.1% formic acid in acetonitrile). The gradient increased from 1 to 9% B over 10 min, followed by 9−19% B over 10 min, 19−32% B over 10 min, 32−48% B over 10 min, 48−58% B over 5 min, 70−95% B over 5 min, isocratic at 95% B for 4 min and a return to 1% B over 1 min.

The eluted peptides were injected into the mass spectrometer operating in V-mode and positive electrospray ionization (ESI) mode with a resolving power of at least 20 000 full-width-at-half-maximum (FWHM). We injected 200 fmol μl^−1^ human Glu-fibrinopeptide B in 1 : 1 (v/v) 0.1% formic acid/acetonitrile at a flow rate of 1 μl min^−1^ via the reference sprayer every 45 s to compensate for mass shifts in MS and MS/MS fragmentation mode. We used data-dependent acquisition (DDA) and data-independent acquisition (DIA), also known as enhanced MS (MS^E^). The DDA cycle consisted of a survey scan covering the *m*/*z* range 400−2000 followed by MS/MS fragmentation of the 10 most intense precursor ions collected at ‘0.2 s’ intervals in the range 50−2000 *m*/*z*. Dynamic exclusion was applied to minimize multiple fragmentations of the same precursor ions. LC-MS^E^ data were collected using alternating low energy (MS) and elevated energy (MS^E^) modes at ‘0.5 s’ intervals in the *m*/*z* range 50−2000 with an interscan delay of 0.05 s. In low-energy mode, data were collected at constant collision energy (4 eV set on the trap T-wave device), which was ramped during the scan from 20 to 45 eV in MS^E^ mode. MS data were collected using MassLynx v. 4.1 (Waters).

### 2.8. Proteomics data processing and protein identification

DDA raw data were used to search a sub-database containing common contaminants (human keratins and trypsin) using ProteinLynx Global Server (PLGS) v. 2.5.2 (Waters) with the following parameters: fixed precursor ion mass tolerance = 10 ppm for the survey peptide, fragment ion mass tolerance = 0.02 Da, estimated calibration error = 0.002 Da, one missed cleavage, fixed cysteine carbamidomethylation and possible methionine oxidation. Unmatched spectra were interpreted de novo to yield peptide sequences for MS-BLAST searches on a local server [24] against an Insecta database (downloaded from NCBI nr on 6 March 2021) and a sub-database obtained by *in silico* translation of the *B. crepitans* transcriptome.

In parallel, pkl files of MS/MS spectra were generated and used to search the NCBI nr database (downloaded on 06 March 2021) combined with *B. crepitans* sub-databases using MASCOT v. 2.5.1 (Matrix Science, UK) with the same parameters as described above. Continuum LC-MS^E^ data were processed using PLGS v. 2.5.2 (Waters). The thresholds for low- and high-energy scan ions and peptide intensity were set to 150, 40 and 1000 counts, respectively. The processed data were used to search the *B. crepitans* protein sub-database combined with a database containing common contaminants (human keratins and trypsin) at a false discovery rate (FDR) of 2%, with the following parameters: fragments per peptide ≥ 3, peptides per protein ≥ 1, fragments per protein ≥ 7 and number of missed tryptic cleavage sites ≤ 1. Searches were restricted to tryptic peptides with fixed cysteine carbamidomethylation and variable methionine oxidation.

#### 2.8.1. Catalase test and thermal resistance of enzymes

Thirty beetles were placed in closed Petri dishes and defensive spraying was triggered by transient exposure to CO_2_ before releasing the beetles back into the terrariums. The secretion was collected with a micropipette and diluted in double-distilled water to a total volume of 200 µl. We then transferred 10 µl to a PCR tube and exposed it to temperatures of 50, 60, 70, 80, 90 and 100°C for 30 s to 12 h using a C100 thermal cycler. The remaining catalytic activity was assessed using the catalase test slide drop method [25] with the following modifications: a 2 µl drop of 30% hydrogen peroxide (Thermo Fisher Scientific) was added to 2 µl of the defensive secretion on a microscope slide. The strength of bubble formation was observed and scored as strong (+++), medium (++), low (+) or no reaction (/) (electronic supplementary material, table S1).

## 3. Results

**Figure 2.**
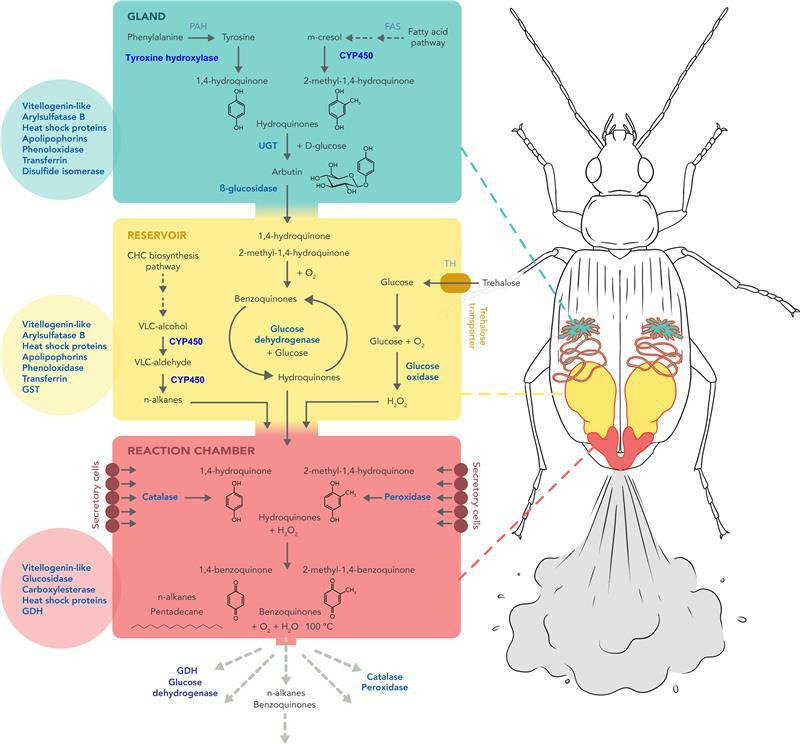
Proposed molecular basis of defensive gland secretions in the bombardier beetle *B. crepitans* based on combined transcriptomic and proteomic analysis. The principal reactions in the MGS with gland (green), RC (yellow) and RXC (red) are shown in black, with candidate genes identified in the transcriptome and the corresponding proteins identified by proteomic analysis shown in blue. The circles show additional abundant proteins identified in the corresponding parts of the defensive gland. More detailed descriptions of the proposed pathways are provided in table 1.

The defensive response of bombardier beetles can be provoked by pinching the legs with forceps (figure 1A). To estimate the time required for recharging, we recorded the spray count and duration in response to stimulation at T_0_ and at hourly intervals thereafter. The glands still produced defensive secretions after 3 h (four rounds of stimulation), but they were depleted after 6 h (seven rounds of stimulation). After a rest period, the spray count and duration in response to stimulation returned to normal, indicating complete replenishment of the MGS after 13 h (electronic supplementary material, figure S1). We therefore assumed that genes required to replenish the explosive chemical reservoirs would be most active after 6 h and chose this stage for the stimulation treatment group (figure 2). Total RNA was isolated from stimulated beetles (stimulation treatment, chemical reservoirs depleted) and controls (no treatment, chemical reservoirs full) to maximize the likelihood of candidate gene identification and to identify genes that are induced or repressed during the recovery phase. We also dissected the entire defensive glands for RNAseq gene expression analysis, as well as separating the MGS/CD, RC and RXC for protein extraction (figure 1C). For proteomic analysis, defensive sprays were also collected in chilled vials containing PBS and protease inhibitors.

**Table 1.** Metabolites involved in the explosive reaction and the candidate genes expressed in *B. crepitans* that may contribute to their synthesis.

metabolites	proposed pathways (genes)	suggested role in *B. crepitans* defensive reaction	localization	*B. crepitans* tissue-specific expression	references
hydroquinones (1,4-hydroquinone, 2-methyl−1,4-hydroquinone)	tyrosine-dependent cuticle tanning pathway via phenylalanine and tyrosine (PAH, TH, PO/laccase, CYP450)	stored precursor metabolites	MGS	yes (PAH, TH, PO, laccase, CYP450)	[15,23–27]
glycosylated hydroquinones	hydroquinone glycosylation pathway (UDP-glycosyltransferase) to generate arbutin; cleavage of arbutin (β-glucosidase)	stable transport form of hydroquinone precursor metabolites	MGS, reservoir	yes (UGT, β-glucosidase)	[27–29]
oxidized hydroquinones (benzoquinones)	reduction of benzoquinones to hydroquinones from glucose requiring glucose dehydrogenase (GDH)	conversion cycle to prevent accumulation of toxic benzoquinones	reservoir	yes (GDH)	[27]
*n*-alkanes (e.g., pentadecane)	fatty acid and cuticular hydrocarbon biosynthetic pathway to form VLC alcohols, VLC aldehydes and *n*-alkanes (FAS, FAR, CYP4G decarbonylase)	alkanes act as organic solvents helping to spread benzoquinones to improve defensive efficiency	reservoir	yes (FAR, CYP450)	[26,30,31]
trehalose and glucose	trehalose stored in haemolymph is converted to glucose (trehalose transporter, trehalase)	required for the generation of hydrogen peroxide	reservoir, surrounding tissues	yes (trehalose transporter, trehalase)	[32]
hydrogen peroxide (H_2_O_2_)	generation of hydrogen peroxide from glucose and O_2_ by glucose oxidase (GO_x_)	hydrogen peroxide required for explosive reaction	reservoir	yes (GO_x_)	[33]
benzoquinones and hydrogen	initiation of explosive reaction with hydroquinones and H_2_O_2_ to produce benzoquinones and H_2_ (catalase, peroxidase)	final explosive reaction to produce heat, water vapour and benzoquinone spray	secretory cells, reaction chamber	yes (catalase, peroxidase)	[13,34]

### 3.1. Transcriptomic analysis

We prepared RNA-Seq libraries from the dissected entire glandular system and remaining body tissues of stimulated and control beetles and constructed de novo transcriptome assemblies for candidate gene identification and differential gene expression analysis. When the differentially expressed genes were ranked by tissue-specific expression patterns (high expression in glands versus body) or fold-change value (stimulated versus control), we found that all the high-ranking genes were strongly expressed or upregulated in the glands of the stimulated beetles and that many encoded unknown proteins, but also enzymes with relevant functions in the generation of explosive secretions (table 1, figure 2; electronic supplementary material, files S1 and S2). For example, we observed the defensive gland-specific expression of genes encoding phenylalanine and tyrosine hydroxylases, phenoloxidases and Cytochrome P450 (CYP450) monooxygenases, which may synthesize 1,4-hydroquinone and 2-methyl-1,4-hydroquinone via the tyrosine-dependent cuticle tanning pathway [15,26,27]. The subsequent glycosylation of these hydroquinones by UDP-glycosyltransferase (UGT) generates arbutin, which is cleaved by β-glucosidase [27–29], and we detected differentially expressed genes encoding both these enzymes identified in the MGS and RC protein extractions. The reduction of benzoquinones to hydroquinones, which prevents the accumulation of these toxic compounds [27], requires GDH and its substrate glucose, which is probably converted from trehalose [32]. Accordingly, a GDH protein was identified in the MGS, and trehalase and a trehalose transporter were found in the RC. Alkanes act as organic solvents that help to spread benzoquinones. The *n*-alkanes in the RC may be produced via the fatty acid and cuticular hydrocarbon biosynthesis pathway, involving the production of very-long-chain alcohols and aldehydes mediated by fatty acyl-coenzyme A reductases and CYP450 [26,30,31]. We found that these proteins were also expressed in the RC. Glucose in the RC is required to generate hydrogen peroxide [33]. A glucose oxidase (GO_x_) that may catalyse this reaction was also expressed specifically in the RC. In the RXC, the explosive reaction of hydroquinones and hydrogen peroxide to produce *p*-benzoquinones and hydrogen may be mediated by catalases and peroxidases [13,34]. Transcriptomic analysis revealed multiple genes specifically expressed in the defensive glands encoding catalases, peroxidases and enzymes involved in the biosynthesis of quinones (figure 2; electronic supplementary material, figures S2 and S3).

### 3.2. Proteomic analysis

**Figure 3.**
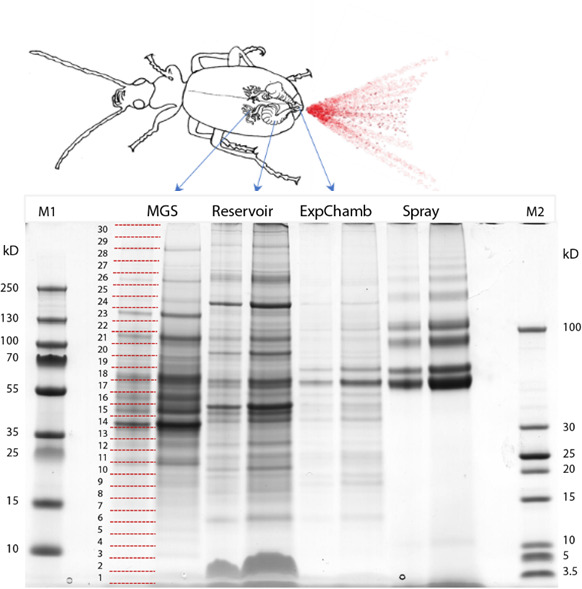
SDS-PAGE analysis of defensive gland fractions and spray proteins from the bombardier beetle *B. crepitans*. The defensive gland was dissected and proteins were extracted separately from the MGS, RC (Reservoir) and RXC (ExpChamb), as well as from the defensive spray (Spray). Following SDS-PAGE, each gel lane was cut into 30 segments (horizontal red lines) for tryptic digestion and proteomic analysis. M1 and M2 are protein marker sets of different sizes, with the indicated molecular masses.

To complement the transcriptomic analysis of the entire gland system, we also separated the MGS/CD, RC and RXC for protein extraction, as well as collecting the defensive spray. The four sets of samples were fractionated by SDS-PAGE, and each gel lane, which showed a unique pattern of bands, was divided into 30 segments for proteomic analysis by LC-MS/MS (figure 3).

The proteomic data confirmed the results of our transcriptomic analysis. The major proteins from each sample revealed the origin of the protein components in the defensive secretions and showed how the defensive response is orchestrated in time and space by the coordinated induction of multiple metabolic pathways (figure 2). Tyrosine hydroxylase was one of the most abundant proteins in the secretory gland tissues (figure 2, MGS) in line with its proposed role to produce 1,4-hydroquinone from phenylalanine, along with a CYP450 monooxygenase that may convert *m*-cresol (3-methylphenol) from the fatty acid pathway into a second hydroquinone compound, 2-methyl-1,4-hydroquinone [15]. The MGS also contained abundant enzymes that allow excess hydroquinones to be stored temporarily as *p*-arbutin, as reported in termites [29], before reconversion to hydroquinones for transfer to the RC. In the RC, stored hydroquinones are slowly oxidized to *p*-benzoquinones. To avoid the unwanted accumulation of these highly reactive and toxic compounds, they are reversibly converted back to hydroquinones in a cyclical reaction involving GDH and glucose [27], the latter mainly derived from trehalose imported via a specific membrane-bound transporter, and most likely converted directly into glucose by trehalase (figure 2, RC). Part of the glucose in the RC is oxidized by GO_x_ to produce large amounts of hydrogen peroxide required for the defensive reaction. The RC also contains various CYP450 monooxygenases that may produce the *n*-alkanes found in the defensive secretions. Finally, the RXC is lined with cells that secrete the catalases and peroxidases identified by transcriptomic analysis, which are responsible for the explosive conversion of hydroquinones and hydrogen peroxide into *p*-benzoquinones plus molecular oxygen and water (figure 2, RXC). The RXC also contains enzymes involved in glucose metabolism (glucosidases and GDH), which may originate from the RC or the secretory cells. The most abundant proteins in all three compartments included vitellogenin-like and heat shock proteins, whereas the MGS and RC also accumulated arylsulfatase B, apolipoproteins, phenoloxidases and transferrin (figure 2; electronic supplementary material, file S3). The catalase and peroxidase genes with the strongest MGS expression profiles were *Bcrep_C163* and *Bcrep_C212*, respectively, according to our transcriptomics data (electronic supplementary material, figure S2). The presence of corresponding enzymes in the RXC and defensive spray was confirmed by LC-MS/MS. Interestingly, the *B. crepitans* peroxidase sequences that were expressed most strongly in the MGS appeared to contain atypical substitutions at the highly conserved haem-binding and substrate-binding sites, suggesting adaptations to fulfil their role in the production of defensive secretions (electronic supplementary material, figure S3).

### 3.3. Catalase test and thermal resistance of enzymes

To evaluate the thermal resistance of the catalase from *B. crepitans*, we mixed the defensive secretions collected from 30 beetles with 30% hydrogen peroxidase at different temperatures for different durations, and used bubble formation to quantify enzyme activity. Even exposure to 90°C for 10 min did not significantly inhibit the formation of bubbles, confirming the outstanding thermal resistance of the catalases responsible for the explosions in the RXC (electronic supplementary material, table S1).

## 4. Discussion

The remarkable defensive strategy of bombardier beetles such as *B. crepitans* has been investigated at the biochemical and physiological levels by a combination of high-speed photography, force analysis and X-ray imaging of the explosive reaction *in vivo*, dissection of the defensive glands, chemical analysis of the spray and stable isotope precursor feeding experiments [10–15]. This revealed the basic mechanistic process and some information about the metabolic requirements: (i) the RC contains hydroquinones and hydrogen peroxide as the precursors of *p*-benzoquinone synthesis [15]; (ii) when the beetle is provoked, muscles surrounding the RC contract and transfer the precursors to the RXC via a one-way valve; and (iii) the presence of catalase and peroxidase in the RXC then causes an exothermic reaction, leading to the explosive release of *p*-benzoquinones, water and oxygen at temperatures of up to 100°C (figure 1A,B) [10–14]. However, it has remained unclear how the hydroquinones are synthesized and safely stored along with the hydrogen peroxide, how the *n*-alkanes also in the RC are synthesized, which peroxidases and catalases trigger the explosive reaction, and how the system recharges after depletion (electronic supplementary material, figure S1).

Our detailed transcriptomic and proteomic analysis has answered many of these outstanding questions. It is now clear that hydroquinones are synthesized in the MGS, presumably via two different pathways: 1,4-hydroquinone from phenylalanine and 2-methyl-1,4-hydroquinone from *m*-cresol, potentially derived from the fatty acid pathway [15]. In the MGS, the hydroquinones are likely to be stored as glycoside derivatives prior to their secretion into the CD reflecting the expression of a glucuronosyltransferase and (to allow the subsequent release of hydroquinones when required) a β-glucosidase before entering the RC. The MGS and RC also contain abundant phenoloxidases. Glucosidases and phenoloxidases, such as tyrosine hydroxylase, are also involved in the production of quinones in the odoriferous glands of the desert stink beetle *Eleodes longicollis* and the red flour beetle *Tribolium castaneum* [35], and at least four glucosidases and five phenoloxidases were identified in the *T. castaneum* stink gland transcriptome [26].

The RXC also contains enzymes involved in glucose metabolism (glucosidases, GO_x_ and GDH), which facilitate the production of hydrogen peroxide and the cyclical interconversion of hydroquinones and *p*-benzoquinones to avoid self-intoxication [27]. The other key pathway in the RXC is cuticular hydrocarbon biosynthesis, producing the cuticular layer that protects the underlying cells from the stored cytotoxic compounds and also leads to the *n*-alkane components that are thought to act as solvents and carriers for the active chemical constituents, promoting spreading and penetration into cuticles. The demand for glucose in the RC is solely met by the abundant trehalose transporter expressed in the RC and the expression of trehalase to release two molecules of glucose from the imported trehalose.

All three compartments express a vitellogenin-like protein, which has also been reported in the ozadene glands of millipedes [36] and the stink gland transcriptome of *T. castaneum* [26]. This protein is thought to promote melanin synthesis in the mealworm *T. molitor*, where *o*-quinones are metabolic intermediates [37]. The MGS and RC also contain arylsulfatase B, which breaks down glycosaminoglycans to release sugars and may transport intermediates in quinone and/or alkane synthesis in *T. castaneum* [26]. Also, arylsulfatase B was recently found to be essential for benzoquinone synthesis in the oral defence secretions of the red palm weevil (*Rhynchophorus ferrugineus*) [38]. The MGS and RC also contain apolipophorins, which may contribute to cuticular hydrocarbon transport and cuticle barrier construction [30]. The presence of transferrin in these compartments is likely to reflect the ability of iron to catalyse reactions involving hydroquinones and hydrogen peroxide [39]. Unsurprisingly, all three compartments of the defensive glands also accumulate abundant heat shock proteins, which act as chaperones to protect proteins by ensuring they are folded correctly under conditions that impose stress, such as high temperatures and exposure to chemicals that promote denaturation [40].

Several pathways and enzymes involved in *n*-alkane and *p*-benzoquinone biosynthesis in the bombardier beetle have also been proposed for the rove beetle (*Dalotia coriaria*), which smears its defensive secretions directly onto attackers [41]. However, a significant difference between the direct release of defensive *p*-benzoquinones and the bombardier beetle’s explosive defence system is the requirement for a highly controlled and spatially separated multi-component system, consisting of hydroquinone precursors, hydrogen peroxide and the activating enzymes (catalase and peroxidase). Although our combined transcriptomic and proteomic approach identified candidate enzymes and pathways that are likely to be involved in the synthesis of defensive compounds, functional studies based on RNA interference or gene knockout are needed to validate these predictions, and fluorescence *in situ* hybridization and/or immunolocalization are needed to confirm the spatial expression of the components at the mRNA and protein levels.

## 5. Conclusion

Our combined transcriptomic and proteomic analysis of the defensive glands in the bombardier beetle *B. crepitans* has enabled the identification of genes and proteins that may contribute to the recharging of the glands following elicited defensive explosions. In line with the predicted functions of the enzymes and transporters, mapping the corresponding protein profiles in each chamber provides a comprehensive spatio-temporal description of how the defence reaction is orchestrated. The unique ability of bombardier beetles to produce chemical explosions thus requires more sophisticated gland architecture at both the anatomical and molecular levels, allowing the spatio-temporal separation of defensive compound synthesis and the independent muscular control of specific glandular parts. These results support our hypothesis that the evolution of explosive secretions was accompanied by the acquisition of thermal resistance by catalases and the functional adaptation of peroxidases involving atypical substitutions in the otherwise highly conserved haem-binding and substrate-binding sites.

## Data Availability

Illumina short reads have been deposited in the EBI short read archive (SRA) with the following sample accession numbers: ERS12235361, ERS12235368–ERS12235370. The complete study can also be accessed directly using the following URL: http://www.ebi.ac.uk/ena/data/view/PRJEB53429. The short-read data and electronic supplemental material, S1, S2 and S3 have been submitted to the Edmond database: [42] (RNA-Seq data), [43] (gland transcriptomic data, annotation and gene expression analysis, electronic supplemental material, S1), [44] (combined transcriptomic data, annotation and gene expression analysis,electronic supplemental File S2) and [45] (proteomics data, electronic supplemental material, S3). Supplementary material is available online [46].
